# *p*-Hydroxycinnamic acid production directly from cellulose using endoglucanase- and tyrosine ammonia lyase-expressing *Streptomyces lividans*

**DOI:** 10.1186/1475-2859-12-45

**Published:** 2013-05-07

**Authors:** Yoshifumi Kawai, Shuhei Noda, Chiaki Ogino, Yasunobu Takeshima, Naoko Okai, Tsutomu Tanaka, Akihiko Kondo

**Affiliations:** 1 Department of Chemical Science and Engineering, Graduate School of Engineering, Kobe University, 1-1 Rokkodai, Nada, Kobe, 657-8501, Japan; 2 Organization of Advanced Science and Technology, Kobe University, 1-1 Rokkodai, Nada, Kobe, 657-8501, Japan; 3 Biomass Engineering Program, RIKEN, 1-7-22, Suehiro-cho, Tsurumi-ku, Yokohama, Kanagawa, 230-0045, Japan

**Keywords:** *Streptomyces lividans*, *P*-hydroxycinnamic acid, Endoglucanase, Cellulose

## Abstract

**Background:**

*p*-Hydroxycinnamic acid (pHCA) is an aromatic compound that serves as a starting material for the production of many commercially valuable chemicals, such as fragrances and pharmaceuticals, and is also used in the synthesis of thermostable polymers. However, chemical synthesis of pHCA is both costly and harmful to the environment. Although pHCA production using microbes has been widely studied, there remains a need for more cost-effective methods, such as the use of biomass as a carbon source. In this study, we produced pHCA using tyrosine ammonia lyase-expressing *Streptomyces lividans*. In order to improve pHCA productivity from cellulose, we constructed a tyrosine ammonia lyase- and endoglucanase (EG)-expressing *S. lividans* transformant and used it to produce pHCA from cellulose.

**Results:**

A *Streptomyces lividans* transformant was constructed to express tyrosine ammonia lyase derived from *Rhodobacter sphaeroides* (RsTAL). The transformant produced 786 or 736 mg/L of pHCA after 7 days of cultivation in medium containing 1% glucose or cellobiose as the carbon source, respectively. To enhance pHCA production from phosphoric acid swollen cellulose (PASC), we introduced the gene encoding EG into RsTAL-expressing *S. lividans*. After 7 days of cultivation, this transformant produced 753, 743, or 500 mg/L of pHCA from 1% glucose, cellobiose, or PASC, respectively.

**Conclusions:**

RsTAL-expressing *S. lividans* can produce pHCA from glucose and cellobiose. Similarly, RsTAL- and EG-expressing *S. lividans* can produce pHCA from glucose and cellobiose with excess EG activity remaining in the supernatant. This transformant demonstrated improved pHCA production from cellulose. Further enhancements in the cellulose degradation capability of the transformant will be necessary in order to achieve further improvements in pHCA production from cellulose.

## Background

In a world in which fossil fuels are becoming scarcer and more expensive, the production of chemicals from renewable feedstocks represents a promising means of meeting energy demands [1-4]. Considering that the market for aromatics is quite large and the chemical synthesis of aromatics is often cumbersome, the bio-based production of these chemicals from renewable resources would provide a green and economically feasible alternative to current technologies [5]. A number of reports have emerged in recent years concerning the production of aromatic compounds using microbes. For example, *Pseudomonas putida* has been used to produce *p*-hydroxystyrene and phenol [6,7], while *p*-hydroxybenzoate production was successfully demonstrated using *Escherichia coli* as a host [8].

The aromatic compound *p*-hydroxycinnamic acid (pHCA) serves as the starting compound for the production of numerous commercially valuable chemicals, including flavors, fragrances, pharmaceuticals, biocosmetics, and health and nutrition products [9], and is also used in the synthesis of thermostable polymers [10]. However, chemical synthesis of pHCA is not cost-effective and results in the production of large amounts of harmful byproducts [11]. Although pHCA production using *P. putida*, *E. coli*, or *Saccharomyces cerevisiae* has been reported [9,11], technical improvements are needed for industrial production.

Tyrosine ammonia lyase (TAL), a member of the aromatic amino acid lyase family, catalyzes the nonoxidative deamination of L-tyrosine to trans-pHCA [12]. Tyrosine ammonia lyase derived from the oleaginous yeast *Rhodotorula glutinis* has been used to produce pHCA by introducing *R. glutinis* TAL into *P. putida*, *E. coli*, and *S. cerevisiae*[9,11]. In addition, *R. glutinis* TAL catalyzes the conversion of L-phenylalanine to trans-cinnamic acid. In this study, we utilized TAL derived from the photosynthetic bacterium *Rhodobacter sphaeroides* (RsTAL) [13]. Because RsTAL demonstrates high substrate specificity for tyrosine, selective production of pHCA is possible.

Cellulose, a β-1, 4-glucose polymer, is the major component of the cell wall of plants and is the most abundant biomass material in the world. The rigid structure of cellulose makes it difficult to degrade into glucose, thus creating a bottleneck in bioprocesses designed to produce useful compounds from cellulose [14,15]. Three types of cellulolytic enzymes are required for the complete breakdown of cellulose to simple sugars. First, endoglucanase (EG) randomly cleaves glycoside bonds within the interior of the cellulose polymer chain. Exocellobiohydrolase (CBH) then acts progressively on the reducing or nonreducing ends of the cellulose chains, releasing either cellobiose or glucose as the major products. Finally, β-glucosidase (BGL) hydrolyzes the cello-oligosaccharides to glucose [16]. By enhancing the expression levels of these three enzymes, effective microbial bioproduction from cellulose can be achieved.

The *Streptomyces* are gram-positive, filamentous bacteria known for their ability to produce various antibiotics and secrete heterologous proteins [17,18]. *Streptomyces lividans* is the most versatile host within this genus for the production of useful compounds. Pagé et al. demonstrated that genetically modified *S. lividans* can secrete a large amount of xylanase in culture [19], while Gamboa-Suasnavart et al. reported production of *Mycobacterium tuberculosis* APA antigen by *S. lividans*[20]. Although *S. lividans* is commonly used for protein production, our previous research involving cinnamic acid production using phenylalanine ammonia lyase-expressing *S. lividans* also showed that *S. lividans* is a suitable host for the production of building block compounds [21], and in the present study we produced pHCA using *S. lividans* as a host. In our previous report, we demonstrated that while *S. lividans* does not completely assimilate cellulose, the organism can assimilate cello-oligosaccharides and glucose [17]. To improve the level of pHCA production from cellulose, in the present study we utilized EG, which catalyzes the degradation of cellulose to cello-oligosaccharide.

In this study, we introduced the gene encoding RsTAL into *S. lividans*, and pHCA was produced from glucose or cellobiose using *S. lividans*/pURsTAL. The production of pHCA when cellobiose served as the carbon source was equal to that obtained with glucose as the carbon source; however, the amount of pHCA produced from cellulose was extremely low. In order to enhance pHCA productivity using cellulose as the carbon source, we therefore constructed an EG- and RsTAL-expressing *S. lividans* strain, and were able to demonstrate for the first time effective production of pHCA directly from cellulose.

## Results and discussion

### Production of pHCA by *S. lividans*/pURsTAL

**Figure 1:**
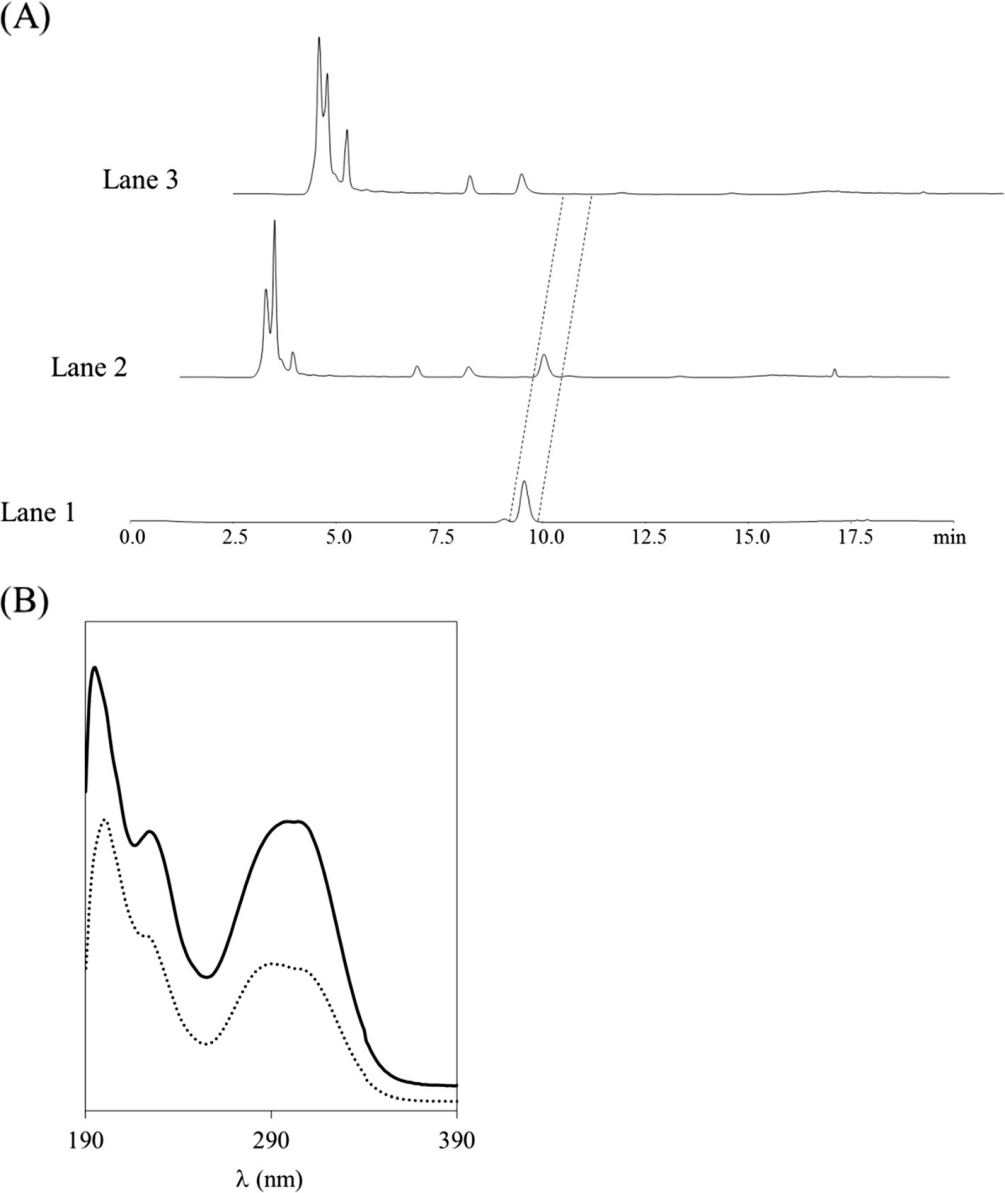
**Confirmation of pHCA produced by RsTAL expressing *S. lividans.*** (**A**) HPLC analysis of pHCA. Lane 1: Standard sample of pHCA in acetonitrile:phosphate buffer (50 mM, pH 2.0) (20:80). Lane 2: *S. lividans*/pURsTAL culture supernatant. Lane 3: *S. lividans*/pU culture supernatant. (**B**) UV spectra of pHCA. Standard sample of pHCA in acetonitrile:phosphate buffer (50 mM, pH 2.0) (20:80) (solid line). The spectrum of the putative pHCA fraction isolated from the *S. lividans*/pURsTAL culture supernatant by HPLC is shown as a dotted line.

*Streptomyces lividans*/pURsTAL and *S. lividans*/pU were cultured separately in TSB medium for 4 days, after which the culture supernatants were analyzed by HPLC. Figure 1A shows representative chromatograms of a standard sample of pHCA solution (Lane 1), *S. lividans*/pURsTAL culture supernatant (Lane 2), and *S. lividans*/pU culture supernatant (Lane 3). In the analysis of the standard solution, the pHCA peak eluted at about 10 min (Lane 1), and a peak eluting with a similar retention time was also observed in the analysis of the *S. lividans*/pURsTAL culture supernatant (Lane 2). In contrast, no pHCA peak was observed on the chromatogram of the *S. lividans*/pU culture supernatant (Lane 3).

A UV analysis of the putative pHCA HPLC fraction isolated from the culture supernatant of *S. lividans*/pURsTAL revealed the presence of three prominent absorption peaks in the 200–350 nm region that were consistent with the peaks produced upon analysis of standard pHCA (Figure 1B). In addition, we carried out pHCA production using *S. lividans*/pURsTAL using the modified TSB medium with 1% glucose and the additional L-tyrosine, which is the precursor of pHCA. The addition of L-tyrosine into the initial culture medium increased the peak areas of pHCA (data not shown). These results confirmed that introduction of the gene encoding RsTAL into *S. lividans* enables the production of pHCA.

**Figure 2:**
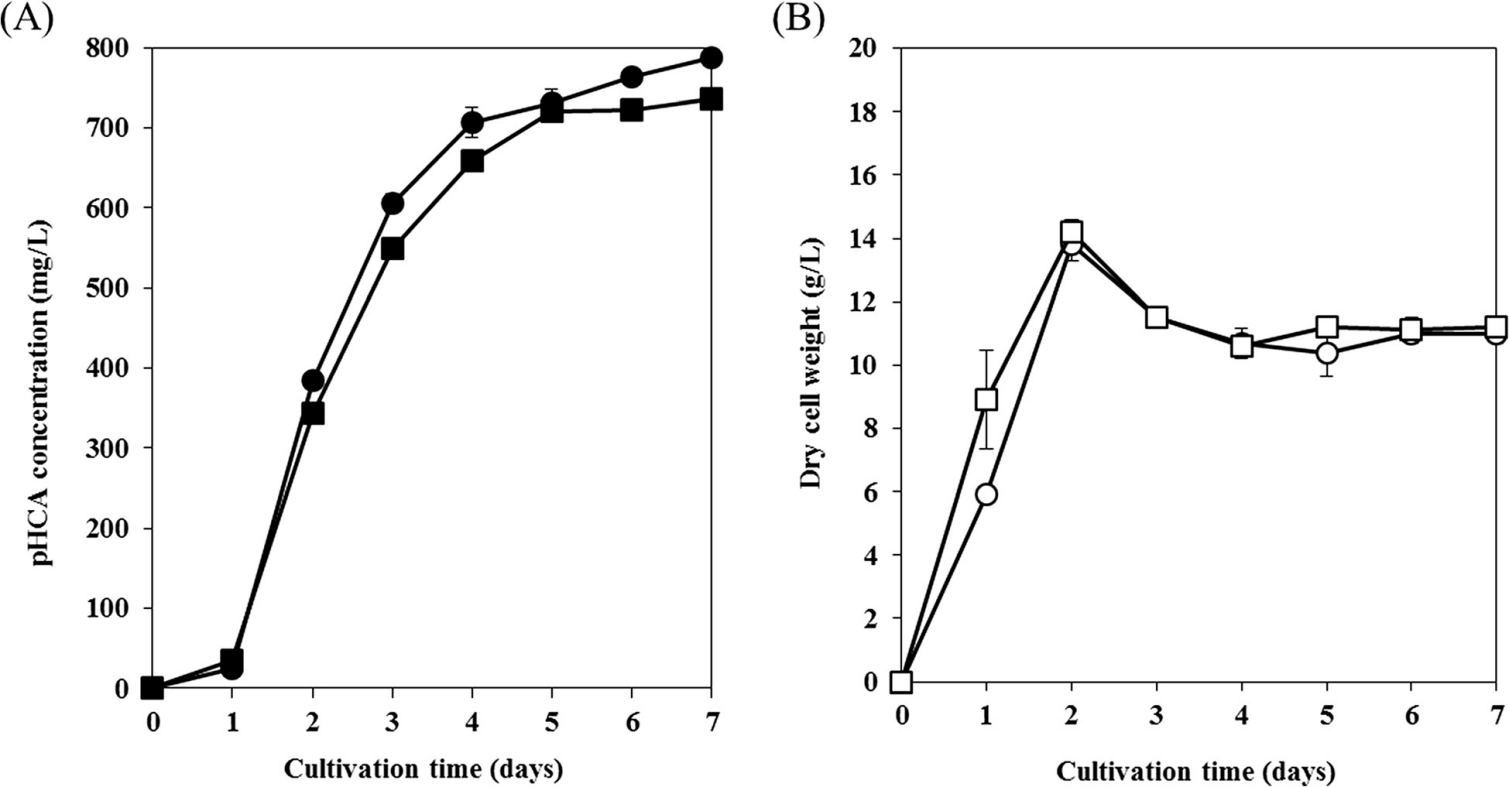
**pHCA production and the cell growth of RsTAL expressing *S. lividans*.** (**A**) Time-course of pHCA production in culture: *S. lividans*/pURsTAL cultured in modified TSB medium containing 1% glucose (closed circles) or 1% cellobiose (closed squares). (**B**) Change in the dry cell weight over time of *S. lividans*/pURsTAL cultured in modified TSB medium containing 1% glucose (open circles) or 1% cellobiose (open squares). Each data point shows the average of three independent experiments, and error bars represent the standard deviation.

In order to demonstrate effective pHCA production, *S. lividans*/pURsTAL was cultured in modified TSB medium with 1% glucose or cellobiose. Figure 2A shows the time courses of pHCA production with each carbon source. The maximum concentration of pHCA produced from glucose and cellobiose was 786 and 736 mg/L, respectively. The level of pHCA produced by *S. lividans* was higher than that reported for batch-cultured *E. coli* (103 mg/L) and *P. putida* S12 (141 mg/L) [9,11]. In the present study, we carried out pHCA production using various concentrations of the additional carbon sources. As a result, 1% carbon sources are suitable for pHCA production using *S. lividans* transformants. In the case of using 1-5% carbon sources, the amount of produced pHCA is almost the same (data not shown). According to these results, we considered that 1% additional carbon sources are suitable for pHCA production using *S. lividans* transformant. In the case of without the additional carbon sources, the amount of produced pHCA was not enough, compared to using the medium with additional glucose or cellobiose.

We also examined pHCA production by the control, *S. lividans*/pU. As expected, this strain did not produce any pHCA (data not shown). Figure 2B shows the change in the dry cell weight over time of *S. lividans*/pURsTAL cultured in medium containing 1% glucose or cellobiose. The growth of *S. lividans*/pURsTAL in medium containing 1% cellobiose as the carbon source was almost the same as in cultures in which 1% glucose served as the carbon source, indicating that *S. lividans* can assimilate cellobiose and produce pHCA as well as utilize glucose, in agreement with our previous report [17]. Here, we estimated the amount of residual glucose or cellobiose using HPLC. In the case of each transformant, glucose or cellobiose added to the medium was consumed within 2 days (data not shown). These results strongly suggested that *S. lividans*/pURsTAL could assimilate cellobiose as well as glucose.

### Construction of EG- and RsTAL-expressing *S. lividans*

Most microorganisms have difficulty degrading cellulose due to its rigid structure. In order to develop effective microbial bioprocesses utilizing cellulose as a carbon source, it is therefore necessary to introduce multiple genes encoding cellulose degradation enzymes into the genome of the organism of interest. A recent report described the introduction of four types of cellulases into *Saccharomyces cerevisiae* and subsequent bioethanol production from cellulose [22]. Bokinsky et al. reported the use of *E. coli* expressing three types of cellulases for the production of advanced biofuels from cellulose [23].

*Streptomyces lividans* constitutively expresses a highly active form of BGL that enables the organism to assimilate cello-oligosaccharide [17]. However, the ability of *S. lividans* to degrade cellulose must be improved in order to achieve effective bioconversion of cellulose to useful compounds. In this study, we demonstrated production of pHCA using cellobiose as the carbon source. To achieve effective pHCA production from cellulose, we then introduced the gene encoding EG into pHCA-producing *S. lividans* in order to facilitate the degradation of cellulose to cello-oligosaccharide. For this purpose we chose Tfu0901, which is a highly active EG derived from *T. fusca* YX, and introduced the gene for this enzyme into RsTAL-expressing *S. lividans*. After the Tfu0901 gene was introduced into wild-type *S. lividans* using the integration type vector pTYM18 [24], the resulting *S. lividans* mutant was able to produce pHCA after introduction of the multicopy type vector pUC702 that carries the gene encoding RsTAL. The resulting EG- and RsTAL-expressing *S. lividans* strain was designated *S. lividans*/pT09pURsTAL.

**Figure 3:**
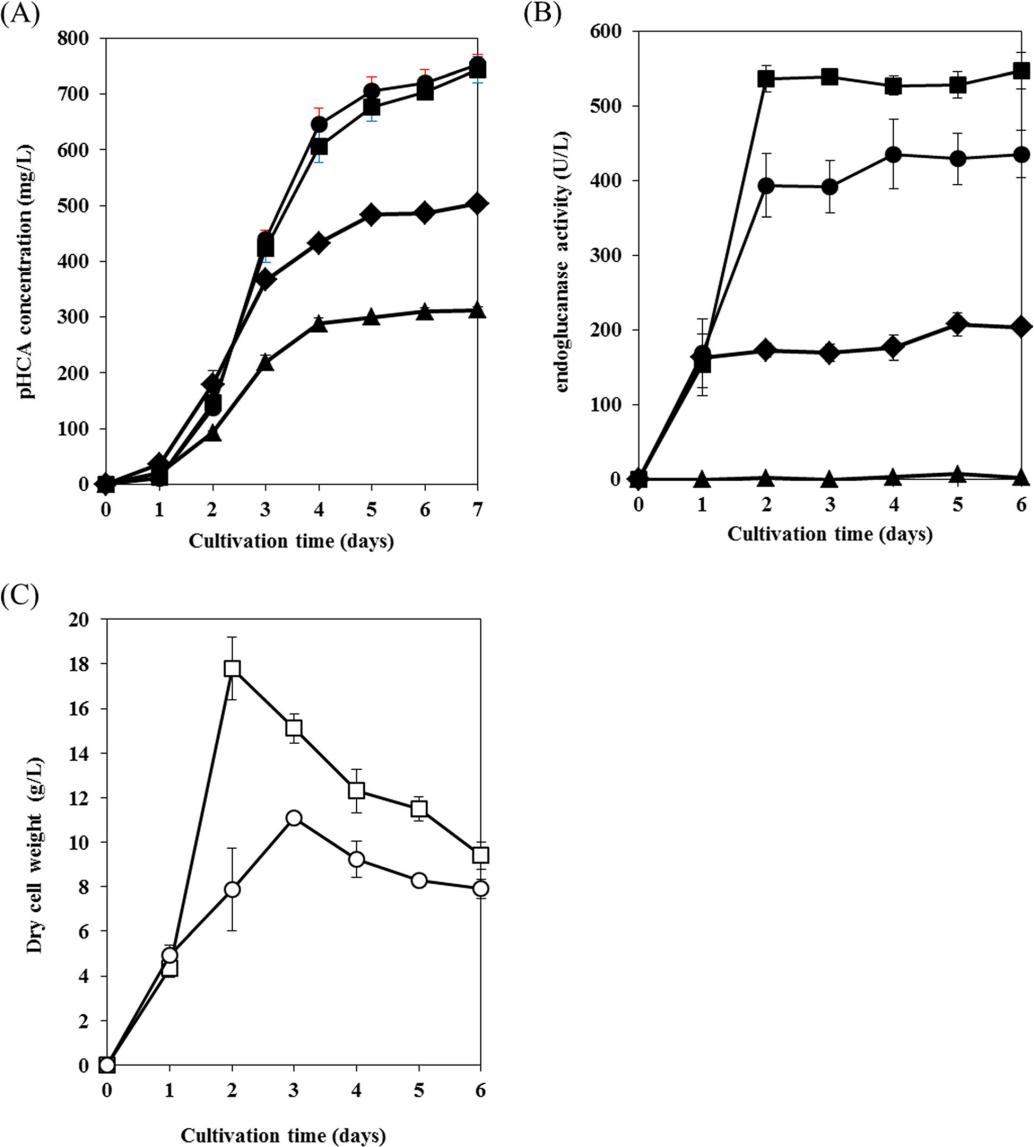
**pHCA production, EG activity and the cell growth of RsTAL and EG expressing *S. lividans.*** (**A**) Time-course of pHCA production in culture: *S. lividans*/pT09pURsTAL cultured in modified TSB medium containing 1% glucose (closed circles), 1% cellobiose (closed squares), or 1% phosphoric acid swollen cellulose (PASC) (closed diamonds); *S. lividans*/pURsTAL cultured in modified TSB medium containing 1% PASC (closed triangles). (**B**) Change in culture supernatant endoglucanase activity over time: *S. lividans*/pT09pURsTAL cultured in modified TSB medium containing 1% glucose (closed circles), 1% cellobiose (closed squares), or 1% PASC (closed diamonds); *S. lividans*/pURsTAL cultured in modified TSB medium containing 1% PASC (closed triangles). (**C**) Change in the dry cell weight over time of *S. lividans*/pT09pURsTAL cultured in modified TSB medium containing 1% glucose (open circles) or 1% cellobiose (open squares). Each data point shows the average of three independent experiments, and error bars represent the standard deviation.

Production of pHCA and expression of EG by *S. lividans*/pT09pURsTAL was examined in modified TSB medium containing 1% glucose. Figure 3A shows the time courses of pHCA production by *S. lividans*/pT09pURsTAL in media containing various carbon sources. Approximately 753 mg/L of pHCA was produced in medium containing 1% glucose. The amount of pHCA produced by *S. lividans*/pT09pURsTAL was equal to that produced by *S. lividans*/pURsTAL, suggesting that EG expression did not affect pHCA production. Figure 3B details the change in supernatant EG activity over time in cultures of *S. lividans*/pT09pURsTAL grown in the presence of various carbon sources. The maximum EG activity of *S. lividans*/pT09pURsTAL cultured with 1% glucose was approximately 440 U/L (Figure 3B), indicating that *S. lividans*/pT09pURsTAL can produce pHCA with EG expressing. The change over time in the dry cell weight of *S. lividans*/pT09pURsTAL cultured with 1% glucose is shown in Figure 3C. The growth of *S. lividans*/pT09pURsTAL in medium containing 1% glucose was similar to that of *S. lividans*/pURsTAL (Figures 2B and 3C), confirming that *S. lividans*/pT09pURsTAL expresses both EG and RsTAL. Here, as shown in Figures 2B and 3C, the decrease of dry cell weight of each *S. lividans* transformant in the stationary phase of the cell growth was confirmed. These results are corresponding to our previous report concerning low-molecular compounds production using *Streptomyces*[17,21].

### Production of pHCA directly from cellulose by EG- and RsTAL-expressing *S. lividans*

Using *S. lividans*/pT09pURsTAL, we produced pHCA from the cellulosic substrate phosphoric acid swollen cellulose (PASC). Figure 3A shows the time course of pHCA production in modified TSB medium containing 1% PASC as the carbon source. The maximal level of pHCA production reached by *S. lividans*/pT09pURsTAL was 500 mg/L after 7 days of cultivation, whereas the control strain, *S. lividans*/pURsTAL, produced 310 mg/L after 7 days of cultivation. The change in supernatant EG activity over time with 1% PASC serving as the carbon source is shown in Figure 3B. The maximal level of EG activity in the supernatant of *S. lividans*/pT09pURsTAL was approximately 210 U/L, whereas that of *S. lividans*/pURsTAL was only 10 U/L. Although *S. lividans*/pURsTAL produced some quantity of pHCA from cellulose, *S. lividans* is known to secrete several kinds of cellulases, including EG, and thus this result is consistent with those of our previous report [17].

In the present study, production of pHCA from cellulose was improved by using the *S. lividans*/pT09pURsTAL strain, which produced 500 mg/L of pHCA, a level that was 1.6-fold higher than the level of pHCA produced by *S. lividans*/pURsTAL. Production of pHCA was carried out using *S. lividans*/pT09pURsTAL cultured with 1% cellobiose as the carbon source. Figure 3A shows the production of pHCA over time in modified TSB medium containing 1% cellobiose as the carbon source. The maximal level of pHCA produced by *S. lividans*/pT09pURsTAL from 1% cellobiose was 743 mg/L after 7 days of cultivation. This level was slightly higher than that produced from 1% PASC (Figure 3A). These results indicate that *S. lividans*/pT09pURsTAL did not completely assimilate the 1% PASC. The amount of pHCA produced by *S. lividans*/pT09pURsTAL from 1% cellobiose was equal to that produced from 1% glucose, and the introduction of the gene encoding EG did not affect the ability of the organism to assimilate cellobiose. Hence, to produce pHCA from cellulose more effectively, the efficiency of the cellulose to cello-oligosaccharide degradation reaction should be improved.

Due to synergism between EG and CBH, cellulose is effectively degraded to cello-oligosaccharide, which is a suitable carbon source for *S. lividans*[22,23]. To enhance pHCA productivity from cellulose, in this study we utilized an EG derived from *T. fusca* YX, Tfu0901, to construct EG- and RsTAL-expressing *S. lividans*/pT09pURsTAL, which was capable of producing pHCA from PASC (Figure 3A) due to the increased availability of cello-oligosaccharides relative to the control strain. However, the amount of pHCA produced from 1% PASC was lower than that produced from 1% glucose or cellobiose (Figure 3A), indicating that PASC was not completely degraded to available sugars. One promising means of achieving higher pHCA productivity from PASC would be to introduce the gene encoding CBH into *S. lividans*/pT09pURsTAL. Exocellobiohydrolase can act on the reducing or nonreducing ends of cellulose chains generated by EG, leading to pHCA production comparable to that obtained when either glucose or cellobiose is employed as the carbon source. Currently, our group is screening for active CBH and developing a three-gene expression system for *S. lividans*.

## Conclusions

We demonstrated the production of pHCA from glucose and cellobiose as carbon sources using RsTAL-expressing *S. lividans*. The amount of pHCA produced in batch culture using our system was higher than that reported in previous studies [9,11]. In order to improve the production of pHCA from cellulose, we constructed a strain of *S. lividans* that expresses both EG and RsTAL. This transformant could secrete EG into the supernatant and produce pHCA directly from cellulose. Our results demonstrate that *S. lividans* can be used as a host to produce aromatic building-blocks from cellulose.

## Methods

### Plasmid construction

The plasmids and primers used in this study are summarized in Table 1. Each polymerase chain reaction (PCR) was carried out using PrimeSTAR HS (Takara, Shiga, Japan). The RsTAL expression vector was constructed as follows. The gene fragment encoding RsTAL was amplified by PCR using the genome of *Rhodobacter sphaeroides* (ATCC 17029) as a template with the RsTAL_F and RsTAL_R primers. The RsTAL fragment was introduced into the *Sph*I and *Nhe*I restriction sites of pUC702-pro-sig-term using an In-Fusion HD Cloning kit (Takara).

**Table 1:** Strains, plasmids, transformants and oligonucleotide primers used in this study

**Strain, plasmid, primer, or transformant**	**Relevant features (5′-3′)**	**Source or reference**
**Strains**		
*Escherichia coli*		
Nova blue	*endA1 hsdR17*(r_*K12*_^*-*^m_*K12*_^+^) *supE44 thi-I gyrA96 relA1 lac* recA1/F^′^[proAB + lacIq ZΔM15::Tn10(Tetr)]	Novagene
S17-1 λpir	*TpR SmR recA*, *thi, pro*, *hsdR*-M^+^RP4: 2-Tc:Mu: Km Tn*7* λpir	BIOMEDAL
*Streptomyces lividans*		
*Streptomyces lividans* 1326	WT strain (NBRC 15675)	NBRC
**Plasmids**		
pUC702-pro-sig-term	Versatile vector for protein expression; thiostrepton resistance marker	Noda et al. 2010
pUC702-*p-*RsTAL	Vector for RsTAL expression; thiostrepton resistance marker	This study
pTYM18	Intergeneric conjugation vector; kanamycin resistance marker	Onaka et al. 2003
pTYM18-pro-sig-term	Versatile integration vector for protein expression; kanamycin resistance marker	This study
pTYM18-*ps-*Tfu0901	Vector for secreting endoglucanase (Tfu0901); kanamycin resistance marker	This study
**Transformants**		
*S. lividans*/pU	Transformant harboring pUC702	This study
*S. lividans*/pURsTAL	Transformant harboring pUC702-*p*-RsTAL	This study
*S. lividans*/pT09pURsTAL	Transformant integrating pTYM18-*ps*-Tfu0901 and harboring pUC702-*p*-RsTAL	This study
**Oligonucleotide primers**		
Tfu0901I_F	aaGCTAGCggtctcaccgccacagtcaccaaag	
Tfu0901I_R	tGGATCCtcagtggtggtggtggtggtgggactggagcttgctccgcacccac	
RsTAL_F	TCGTTTAAGGATGCAatgaagccaatgctcgccat	
RsTAL_R	CGCTCAGTCGTCTCAgctgatcgccatcgaggtc	

The integration-type vector for the expression of EG was constructed as follows. First, pUC702-pro-sig-term was digested with *Hind*III and *Kpn*I, and the resulting fragment encoding the promoter, signal sequence, and terminator was subcloned into the *Hind*III and *Kpn*I restriction sites of pTYM18 [24,25], which served as a shuttle vector between *E. coli* and *S. lividans*. The resulting plasmid was designated pTYM18-pro-sig-term. The gene fragments encoding Tfu0901 were amplified by PCR using the *T. fusca* YX genome (ATCC 27730) as a template with the Tfu0901I_F and Tfu0901I_R primers. The Tfu0901 fragment was digested with *Nhe*I and *Bam*HI and introduced into the *Nhe*I and *Bam*HI restriction sites of pTYM18-pro-sig-term.

### Bacterial strains, transformation, and cultivation

The strains and transformants used in this study are summarized in Table 1. The integration-type plasmid pTYM18-*ps*-Tfu0901 was constructed and transformed into *E. coli* S17-1 λpir (BIOMEDAL, Seville, Spain). A single transformant colony was picked and cultivated at 37°C for 8 h in 5 mL of LB medium containing 40 μg/mL of kanamycin. The cells were harvested and washed three times with LB broth to remove the kanamycin. Next, the cells were suspended in 500 μL of LB medium and mixed with wild-type *S. lividans* spores. The mixture was plated on ISP4 medium (1.0% soluble starch, 0.1% K_2_HPO_4_, 0.1% MgSO_4_ · 7 H_2_O, 0.1% NaCl, 0.2% (NH_4_)_2_SO_4_, 0.2% CaCO_3_, 0.0001% FeSO_4_, 0.0001% MnCl_2_, 0.0001% ZnSO_4_, and 2.0% agar) and incubated for 18 h at 30°C. A 3-mL aliquot of soft-agar nutrient broth containing kanamycin (50 μg/mL) and nalidixic acid (67 μg/mL) was dispensed in layers onto the plate, which was then incubated at 30°C for 5 days, after which a single colony was picked and streaked onto ISP4 agar containing kanamycin (50 μg/mL) and nalidixic acid (5 μg/mL) and incubated at 30°C for 5 days for screening.

Protplasts of wild-type *S. lividans* 1326 and *S. lividans*/pT09 were prepared according to the method of Hopwood et al. [26]. Briefly, mycelia of each strain were treated with 1 mg/mL of lysozyme solution (Wako, Osaka, Japan), and the suspended mycelia were used as protoplasts. The plasmid pUC702-*p*-RsTAL was introduced into wild-type *S. lividans* using the polyethylene glycol method. The pUC702-*p*-RsTAL plasmid was also introduced into *S. lividans*/pT09. Selection of transformants was carried out by overlaying soft agar containing 50 μg/mL of thiostrepton, or 50 μg/mL of thiostrepton and kanamycin.

To produce pHCA, a single colony of either *S. lividans*/pURsTAL or *S. lividans*/pT09pURsTAL was inoculated into a test tube containing 5 mL of TSB medium supplemented with 5 μg/mL of thiostrepton or 5 μg/mL of thiostrepton and 50 μg/mL of kanamycin, followed by cultivation at 28°C for 3 days. Next, 5 mL of *S. lividans*/pURsTAL or *S. lividans*/pT09pURsTAL preculture medium was seeded into a shake flask equipped with a baffle and containing 100 mL of modified TSB medium with 1% glucose, cellobiose, or PASC as a carbon source, and the flask was incubated at 28°C for 7 days. In this study, modified TSB medium consisted of 3% TSB with 5% tryptone as the nitrogen source.

### Measurement of EG activity

Endoglucanase activity was measured according to a method established by Miller [27], with some modifications. Briefly, a 300-μL aliquot of culture supernatant was mixed with 700 μL of a 1% (w/v) solution of carboxymethylcellulose (CMC) dissolved in 50 mM sodium acetate buffer (pH 7.0) and the mixture was incubated at 50°C for 1 h. The amount of reducing sugar released from the CMC was assessed by determining the amount of glucose and equivalents present using the dinitrosalicylic acid method. One unit of enzyme activity was defined as the amount of enzyme required to release 1 μmol of reducing sugar as glucose, for CMC, and equivalents, from the substrate, per min.

### Analytical methods

The pHCA concentration was determined by high-performance liquid chromatography (HPLC; Shimadzu, Kyoto, Japan) using a Cholester column (Nacalai Tesque, Kyoto, Japan). The HPLC column was maintained at 30°C and eluted at a flow rate of 1.2 mL/min. A dual solvent system was used. Solvent A was phosphate buffer (50 mM, pH 2.0) and solvent B was acetonitrile. The gradient began at 80% solvent A and 20% solvent B, and a 50–50 mixture of solvents A and B was employed from 12 to 17 min. From 17.01 to 20.00 min the solvent composition was 80% solvent A and 20% solvent B.

The concentration of pHCA was determined using an ultraviolet absorbance detector (SPD-20AV, Shimadzu). Culture supernatant was obtained by centrifuging the culture broth at 21,880 × g for 20 min, and was followed by HPLC analysis of the supernatant. A JASCO V-650 spectrophotometer (JASCO Corporation, Tokyo, Japan) was used to collect UV absorption spectra.

## Competing interests

The authors declare that they have no competing interests.

## Authors’ contributions

YK and SN designed the experiments. YK performed the experiments. YK, SN and TT wrote the paper. CO, YT and NO provided the genome of *Rhodobacter sphaeroides* (ATCC 17029). AK commented and supervised on the manuscript. All authors approved the final manuscript.
